# Genome Organization of Canada Goose Coronavirus, A Novel Species Identified in a Mass Die-off of Canada Geese

**DOI:** 10.1038/s41598-019-42355-y

**Published:** 2019-04-11

**Authors:** Amber Papineau, Yohannes Berhane, Todd N. Wylie, Kristine M. Wylie, Samuel Sharpe, Oliver Lung

**Affiliations:** 1 National Centre for Foreign Animal Disease, Canadian Food Inspection Agency, Winnipeg, MB Canada; 2 Department of Biological Sciences, University of Manitoba, Winnipeg, MB Canada; 3 Department of Pediatrics, Washington University School of Medicine, St. Louis, St. Louis, MO USA; 4 McDonnell Genome Institute, Washington University School of Medicine in St. Louis, St. Louis, MO USA; 5 Faculty of Veterinary Medicine, University of Calgary, Calgary, AB Canada

## Abstract

The complete genome of a novel coronavirus was sequenced directly from the cloacal swab of a Canada goose that perished in a die-off of Canada and Snow geese in Cambridge Bay, Nunavut, Canada. Comparative genomics and phylogenetic analysis indicate it is a new species of *Gammacoronavirus*, as it falls below the threshold of 90% amino acid similarity in the protein domains used to demarcate *Coronaviridae*. Additional features that distinguish the genome of Canada goose coronavirus include 6 novel ORFs, a partial duplication of the 4 gene and a presumptive change in the proteolytic processing of polyproteins 1a and 1ab.

## Introduction

Viruses belonging to the *Coronaviridae* family have a single stranded positive sense RNA genome of 26–31 kb. Members of this family include both human pathogens, such as severe acute respiratory syndrome virus (SARS-CoV)^1^, and animal pathogens, such as porcine epidemic diarrhea virus^2^. Currently, the International Committee on the Taxonomy of Viruses (ICTV) recognizes four genera in the *Coronaviridae* family: *Alphacoronavirus, Betacoronavirus, Gammacoronavirus and Deltacoronavirus*. While the reservoirs of the *Alphacoronavirus* and *Betacoronavirus* genera are believed to be bats, the *Gammacoronavirus* and *Deltacoronavirus* genera have been shown to spread primarily through birds^3^. The first three species of the *Deltacoronavirus* genus were discovered in 2009^4^ and recent work has vastly expanded the *Deltacoronavirus* genus, adding seven additional species^3^.

By contrast relatively few species within the *Gammacoronavirus* genus have been identified. There are currently two recognized species in the *Gammacoronavirus* genus: avian coronavirus (ACoV) and beluga whale coronavirus SW1 (SW1). ACoVs infect multiple avian hosts and include several important poultry pathogens, such as infectious bronchitis virus (IBV) and turkey coronavirus (TCoV)^5^. IBV was first described in the United States^6^ but has since been described around the globe^7^. Turkey Coronavirus is the cause of acute enteritis in domestic turkeys^8^. The second species in the *Gammacornavirus* genus SW1 was first discovered in beluga whales^9^ but has since been detected in other cetaceans, such as Indo-Pacific bottlenose dolphins^10^. Despite IBV being the first discovered coronavirus and the impact it has on the poultry industry^11^, the number of identified species within the *Gammacoronavirus* genus remains small in comparison to the other coronavirus genera. Coronaviruses from several other avian hosts for which partial sequences are available suggest relatedness to IBV and TCoV. These viruses, which include goose coronavirus (GCoV), were tentatively classified as part of the ACoV species. An approximately 3 kb region, including the nucleocapsid gene and several accessory genes, of GCoV were previously sequenced from a greylag goose in Norway^12^.

Here we present the full genome of Canada goose coronavirus (CGCoV) sequenced directly from the cloacal swab of a Canada goose, which expired in a mass die-off in a remote region near the arctic in Nunavut, Canada. Our analyses demonstrate that it should be classified as a novel species in the *Gammacoronavirus* genus.

## Results and Discussion

**Figure 1:**
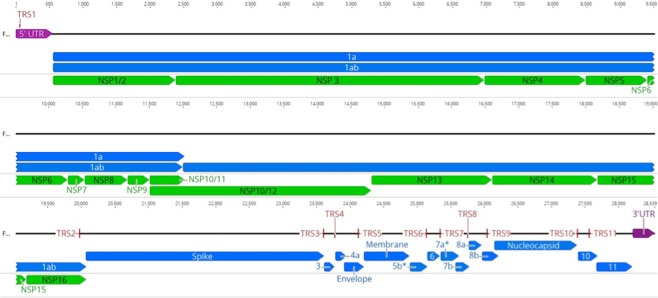
Genome organization of Canada goose coronavirus. Purple indicates untranslated regions, blue indicates putative proteins, green indicates coding region of mature non-structural proteins (NSP) and red indicates transcription regulatory sequences (TRS). The stem loop-like motif and octamer motif are contained within the 3′ UTR. Genome organization figure was constructed using Geneious^TM^ (Biomatters, v 9.1.8). *Indicate ACoV 4b homologues. Proteins are named numerically from the 5′ end of the genome, with the exception of the structural genes, which are denoted by their common names.

Due to the remote location of the die off, samples from the dead birds were not collected immediately and sent to a diagnostic laboratory until severe predation and decomposition had occurred. The poor sample quality, in addition to the difficulty of coronavirus isolation, led to the failure to isolate infectious virus using standard methods. However, the complete genome of a novel gammacoronavirus was assembled from high throughput sequencing reads derived from the cloacal swab of a single Canada goose. The assembled genome of the novel Canada goose coronavirus (CGCoV) is 28,539 nts in length (excluding the poly(A) tail) and has 38.4% GC-content. The genome of CGCoV is approximately 1000 nts longer than the reference genomes for ACoV available in GenBank. The genome organization of CGCoV is presented in Fig. 1. The 5′ UTR of CGCoV is 553 nt in length and contains a higher GC content (48.3%) relative to the genome as a whole. The 5′ UTR of CGCoV shares only 68% pairwise identity with that of duck coronavirus (DCoV) and 47.5% pairwise identity to that of SW1. Like all coronavirus genomes reported to date, CGCoV’s genome is dominated by the coding regions for the large polyproteins 1a and 1ab, followed by the structural and accessory genes. The heptanucleotide slippery sequence UUUAAAC, associated with the ribosomal slippage that produces polyprotein 1ab, was present at nt positon 11,995. CGCoV’s genome contains genes for all four structural proteins common to coronaviruses; spike (S), envelope (E), membrane (M) and nucleocapsid (N). In addition, CGCoV contains 10 open reading frames (ORFs) predicted to encode accessory proteins. The order of the structural and accessory protein-coding ORFs in CGCoV resembles that of ACoV, but there are notable differences. The general genome organization of ACoV is 1ab-S-3a-3b-E-M-4b-4c-5a-5b-N-6b^13^. However, there is some variance in the genome organization within the ACoV species. For example, Australian IBV strains lack ORFs 4a, 4b and 5b^14^. Overall, CGCoV contains a larger number (n = 14) of ORFs coding for predicted accessory and structural proteins downstream of the polyprotein 1ab coding region. Two additional ORFs (7a and 7b) are found between the CGCoV M and N ORFs. There are also two additional ORFs (10 and 11) following the N gene. While some ACoVs do have ORFs following the N gene, ORFs 10 and 11 in CGCoV do not share obvious homology to those of IBV and TCoV. The 3′ UTR of CGCoV is 301 nucleotides in length and contains the stem loop-like motif 113 bp upstream from the poly(A) tail. This stem loop-like motif was first identified in astroviruses^15^ but is also present in ACoVs and SARS-CoV^13^. Further downstream in the 3′ UTR, the octanucleotide motif (GGAAGAGC) is found 71 bp upstream of the poly(A) tail. The 3′ UTR of CGCoV shares 98% pairwise identity to the partially sequenced GCoV and 84% pairwise identity to IBV.

A trait suggesting common ancestry between CGCoV and ACoV is the canonical ACoV transcription regulatory sequence (TRS) found at the end of the leader sequence in CGCoV. The TRS of CGCoV is identical to that identified by Cao *et al*. (2008) as the TRS of TCoV (CTTAACAAA). Body TRS’s regulate viral gene expression by forming a complex with the leader TRS, causing discontinuous transcription of mRNA^16^. Ten putative body TRSs were found in the 3′ end of the CGCoV genome (Fig. 1). Four of the ten putative TRSs (4, 6, 8, 9) were exact matches to the canonical leader TRS. Three TRSs (2, 7, 11) contained one mismatch and the remaining three TRSs (3, 5, 10) contained two mismatches to the leader TRS. The functionality of these TRSs would need to be experimentally determined; however, previous studies have shown that TRSs of ACoVs are subject to some variation^13,17^. CGCoV contains twice the number of TRS’s as ACoVs and a similar number compared to the nine contained in SW1^9^. Table 1 demonstrates the nucleotide distances between the TRS and the start codon of ORFs found in CGCoV’s, which are comparable to those of TCoV^13^.

**Table 1:** Putative viral proteins of Canada goose coronavirus.

Protein	Top Match in NCBI	Top match - aa % identity*	Size (aa)	Distance between TRS and start codon (nt)
1a	1a-Infectious bronchitis virus strain B1648	43	3825	480
1ab	1ab-Infectious bronchitis virus strain ck/CH/LJL/05I	57	6510	480
Spike	Spike-Infectious bronchitis virus strain N2-75	53	1184	82
3	n/a	n/a	53	0
4a	n/a	n/a	55	3
Envelope	Envelope-Infectious bronchitis virus strain IS-1494	69	100	n/a
Membrane	Membrane-Duck Coronavirus isolate DK/GD/2014	72	235	74
5b	4b-Infectious bronchitis virus strain Georgia 1998 Vaccine	41	88	n/a
6	n/a	n/a	63	5
7a	4b-Duck Coronavirus isolate DK/GD/2014	23	92	3
7b	n/a	n/a	69	n/a
8a	5a-Duck Coronavirus isolate DK/GD/2014	37	65	4
8b	5b-Duck Coronavirus isolate DK/GD/2014	46	85	n/a
Nucleocapsid	Nucleocapsid-Goose Coronavirus	94	414	94
10	ORFxg-Goose Coronavirus	92	97	0
11	ORFyg-Goose Coronavirus	81	180	91

*Matches below 20% coverage not shown.

The start codon of CGCoV’s polyprotein 1ab is located 567 nucleotides downstream of the leader TRS. The coronavirus polyprotein 1ab is cleaved into 15–16 non-structural proteins (NSPs) by two viral proteases^18^. Putative cleavage sites for these proteases are present in CGCoV’s 1a and 1ab polyproteins, with the exception of the NSP 10/11 (polyprotein 1a) and NSP 10/12 (polyprotein 1ab) cleavage sites. The missing cleavage site would be located near the end of polyprotein 1a, producing the NSPs 10 and 11, and also in the alternatively transcribed polyprotein 1ab, producing NSPs 10 and 12. The absence of the NSP10/11 and 10/12 protease recognition site was confirmed with Sanger sequencing. With the exception of the missing cleavage sites, the putative cleavage sites would produce NSPs of sizes congruent with other *Gammacoronavirus* species (Table 2). No *Gammacoronavirus* species to date, including CGCoV, have a papain-like protease cleavage site between NSP 1-2^19^.

**Table 2:** Non-structural proteins size and cleavage site of gammacoronaviruses.

Protein	CGCoV		TCoV		IBV		SW1	
	Cleavage site	Size aa	Cleavage site	Size aa	Cleavage site	Size aa	Cleavage site	Size aa
NSP1/2	AG^GH	609	AG^GK	673	AG^GK	673	VD^GD	636
NSP3	AG^GV	1532	AG^GV	1594	AG^GI	1592	LG^GV	1586
NSP4	LQ^AG	503	LQ^AG	514	LQ^SG	514	LQ^AG	537
NSP5	LQ^SN	307	LQ^SS	307	LQ^SS	307	LQ^SN	303
NSP6	VQ^SK	295	VQ^SK	297	VQ^AK	293	VQ^SK	303
NSP7	LQ^AV	83	LQ^SV	83	LQ^SV	83	LQ^AV	83
NSP8	LQ^NN	212	LQ^NN	210	LQ^NN	210	LQ^NN	198
NSP9	LQ^GK	111	LQ^SK	111	LQ^SK	111	LQ^HG	112
NSP10	SRFV*	**173**	**VQ^SA**	**145**	**VQ^SV**	**145**	**LQ^SV**	**189**
NSP11	**—**	**—**	**—**	**23**	**—**	**23**	**—**	**17**
NSP12	SRFV*	**1101**	**VQ^SA**	**941**	**VQ^SV**	**940**	**LQ^SV**	**926**
NSP13	LQ^SC	599	LQ^SC	601	LQ^SC	600	LQ^AS	601
NSP14	LQ^SN	522	LQ^GT	521	LQ^GT	514	LQ^SQ	528
NSP15	LQ^SI	338	LQ^SI	338	LQ^SI	338	LQ^SL	349
NSP16	LQ^SG	298	LQ^SA	302	LQ^SA	302	LQ^SD	312

*Amino acids present in CGCoV where putative protease cleavage sites were observed in TCoV, IBV and SW1.

While the genome structure of CGCoV resembles that of ACoV, there are some notable differences. For example, there are no homologues to ACoV’s 3a or 3b accessory proteins in CGCoV, a trait shared with SW1. Furthermore, CGCoV has a number of ORFs that do not appear to have homologues in other sequenced *Gammacoronavirus* species, such as the ORFs for putative proteins 3 and 4a (Fig. 1). These two ORFs are found in CGCoV in the corresponding location of ACoV’s 3a and 3b ORFs (between the S and E ORFs) and are also similar in size to ACoV’s 3a and 3b proteins. However, they share no obvious sequence similarity with any 3a or 3b gene, or any other entry in NCBI (Table 1). ACoV’s 3a and 3b proteins have been shown to be unnecessary for replication^20^, however knock-out mutants for these accessory genes are attenuated^21^. The IBV’s 3 gene is functionally tricistronic, meaning the 3a, 3b and E proteins are under the control of a single TRS^22,23^. This is not the case in CGCoV, as the E ORF of CGCoV shares a TRS with only the 4a ORF in CGCoV and 3 ORF is preceded by a separate TRS (Fig. 1).

An additional TRS is also found in between CGCoV’s M and N ORFs, preceding the proteins 7a and 7b (Fig. 1). Commonly ACoV’s have two ORFs between the M and 5 genes, coding for the 4b and 4c accessory proteins. CGCoV contains 4 ORFs between the M and 8 gene (ACoV 5 gene homologue). Two of these ORFs (5b and 7a) are ACoV 4b homologues, likely the result of gene duplication. This area in IBV has been identified as a hotspot for recombination^24^. The region between the ACoV M and 5 gene was formally called the intergenic region because of the lack of a TRS. However, it was later shown that gene 4 is expressed using an alternative TRS in IBV^17^. Notably, one of the 4b homologs (i.e. 5b) in CGCoV does have a TRS (Fig. 1). The use of template switching at TRSs is thought to lend to recombination in coronaviruses^25^. The two CGCoV 4b homologs are not identical to each other (Table 1). Amino acid sequence identity to other 4b proteins is low for both CGCoV 4b homologues, 41% to IBV and 23% to DCoV respectively. The gene 4 duplication was also confirmed by Sanger sequencing of the genomic region between the M ORF to the 8 gene.

The ACoV 5a and 5b accessory proteins (8a and 8b in CGCoV) appear to be the only accessory proteins conserved in all 3 *Gammacoronavirus* species, although gene order differs. ORFs encoding putitive proteins 5a and 5b belong to the bicistronic gene 5 of ACoVs and are also unnecessary for replication^21^. To date, all publically available sequence information suggest that *Gammacoronavirus* species have lost the NSP1 cleavage site. The function of NSP1 in alphacoronaviruses and betacoronaviruses is the inhibition of host protein production. Accessory protein 5a is shown to have adopted this function in place of NSP1 in IBV^19^.

**Figure 2:**
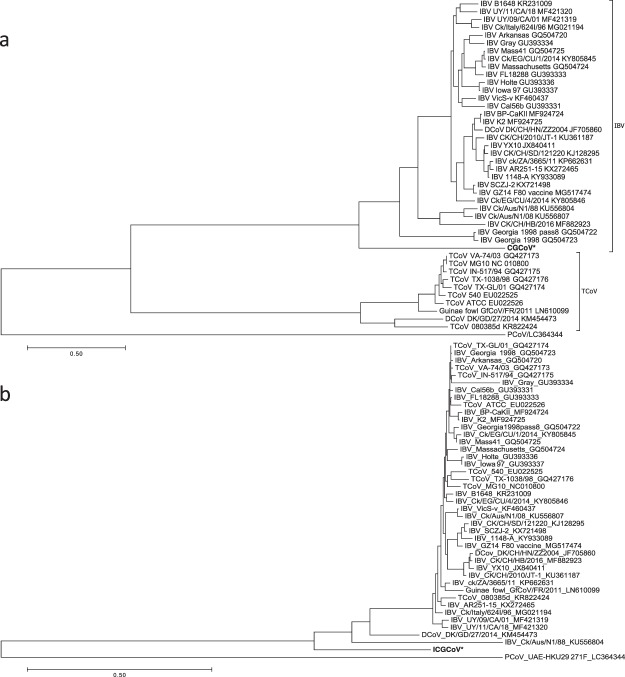
The phylogeny of gammacoronavirus spike and nucleocapsid proteins. A maximum likelihood tree built, using the amino acid sequences of the spike protein (**a**) and nucleocapsid protein (**b**) domains aligned with ClustalW^31^, in MEGA X using the Jones-Taylor-Thornton (JTT) substitution model and 1000 bootstraps^32^. IBV Infectious Bronchitis virus, TCoV Turkey Coronavavirus, PCoV Pigeon Coronavirus, DCoV Duck Coronavirus.

The majority of structural proteins of CGCoV also share low amino acid sequence identity (53–72%) with IBV and DCoV. Phylogenetic analysis of the spike gene show that the CGCoV spike gene clusters with the IBV spike gene, separate from the TCoV cluster (Fig. 2a). Figure 2b also demonstrates the nucleocapsid gene of CGCoV is distantly related to those of ACoVs. However the CGCoV nucleocapid protein does share 94% amino acid sequence identity with the nucleocapsid protein encoded in the partially sequenced graylag GCoV genome^13^. In addition, ORFs 10 and 11, which are preceded by the nucleocapsid gene, also share high amino acid identity with graylag GCoV proteins, 92% and 81% respectively. It should be noted that, among full and partial genomes of gammacoronaviruses sequenced to date, ORFs 10 and 11 seem to be unique to CGCoV and GCoV and are both preceded by a TRS, suggesting that these ORFs are very likely expressed. The fact that some CGCoV proteins share higher amino acid sequence similarity with the partial GCoV sequences available suggest these two viruses are more closely related to each other than to other gammacoronaviruses known to date.

**Figure 3:**
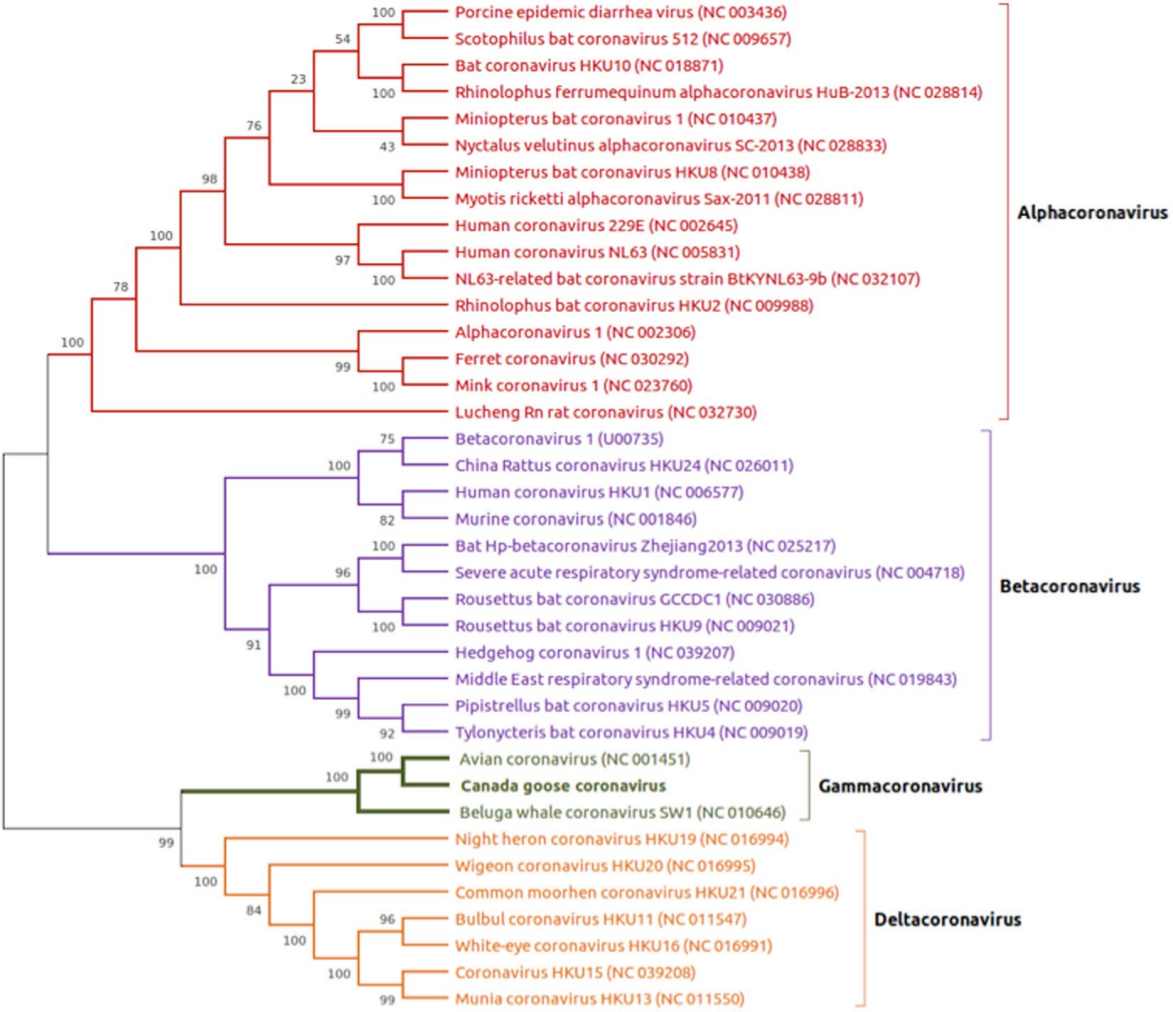
The phylogeny of Canada goose coronavirus. A maximum likelihood tree built, using the concatenated amino acid sequences of the replicase and helicase protein domains aligned with ClustalW^31^, in MEGA X using the Jones-Taylor-Thornton (JTT) substitution model and 1000 bootstraps^32^. Numbers at nodes indicate the bootstrap value.

The phylogenetic tree built using the coding regions for the conserved replicase and helicase domains demonstrates that CGCoV clusters with gammacoronaviruses and shares a more recent common ancestor with ACoV than with the cetacean gammacoronaviruses (Fig. 3). Further comparisons suggest that CGCoV is a separate species from ACoV. Current taxonomy of *Coronaviridae* is determined using pairwise comparisons of the amino acid sequence of seven conserved domains in the 1ab polyprotein. Members of the same species share over 90% amino acid identity in these seven conserved domains^5^. Percent identity of CGCoV falls well below the 90% threshold set by ICTV with ACoV and SW1, suggesting CGCoV is a separate species (Table 3). Within *Coronaviridae*, CGCoV shares the highest homology (68%) in the 7 conserved domains to the gammacoronaviruses TCoV and DCoV.

**Table 3:** Comparison of the amino acid pairwise identity of 7 conserved coronavirus domains in the poly1ab protein of Canada goose coronavirus to other gammacoronaviruses.

Domain	aa % identity to IBV	aa % identity to TCoV	aa % identity to DCoV	aa % Identity to SW1
ADP-ribose-1″-phosphatase	42	43	38	23
3C-like Protease	56	58	57	49
RdRp	80	80	83	69
Helicase 1	89	90	92	78
Exonuclease	78	72	77	56
Endoribonuclease	53	53	54	41
Ribose-2′-O methyltransferase	74	77	76	65
Average	67	68	68	54

As the full genome was sequenced from only the cloacal swab of a single Canada goose, a screening PCR was designed based on the 4b duplication region unique to CGCoV and performed on all samples. The Sanger sequencing primers of the region between the M and 8 gene were used, as this area of the genome is specific to CGCoV. All samples were found to be positive, with the exception of the pharyngeal swab of the snow goose and the lung tissue of the second Canada goose which could not be tested as the sample was exhausted. Amplicons were Sanger sequenced and confirmed to match the CGCoV genome. High throughput sequencing conducted on RNA extracted from cloacal swabs from the second Canada goose and the snow goose also resulted in partial (64 and 18%) genomes of the CGCoV. While this does not confirm the virus’s presence in all animals that perished in the die off, this shows CGCoV was present in all birds that were available for testing. Further studies will require the availability of an infectious virus to determine the pathogenicity of CGCoV and its ability to cause mortality in Canada geese and snow geese.

To summarize, the complete genome of CGCoV, a novel *Gammacoronavirus* species was sequenced directly from the cloacal swab of a Canada goose associated with a mass die-off. The CGCoV genome was also detected in samples derived from a second Canada goose and a snow goose that perished in the die-off, using PCR, Sanger and high throughput sequencing. Comparative genomics and phylogenetic analysis indicate CGCoV clusters with ACoV but is a distinct *Gammacoronavirus* species. Interesting features of this new species include the presence of two 4b homologues, a putative change in the proteolytic processing of the polyproteins 1a and 1ab, and six novel accessory genes.

## Methods

### Source of samples

A large die off of Canada and snow geese occurred in the fall of 2017 near the arctic in Cambridge Bay, Nunavut, Canada. Due to poor carcass quality and remote location, samples were only collected from two dead Canada geese and one Snow goose, all of which had undergone predation and decomposition. Cloacal and pharygenal swabs were collected from all three birds, lung tissue was collected from one Canada goose. Other organs were not present or were in extremely poor condition. Detection of both common avian pathogens, such as avian influenza and avian paramyxovirus by the National Reference Laboratory, by routine laboratory testing gave negative results. Virus isolation was performed by two serial passages in SPF chicken eggs using protocols prescribed by the World Organization for Animal Health (OIE) for the most closely related gammacoronavirus, infectious bronchitis virus (IBV). Samples were then subjected to targeted sequence enrichment^26^ and next-generation sequencing on an Illumina MiSeq platform.

### Sample pre-treatment

Tissues were homogenized using a Precellys Evolution homogenizer (Bertin Instruments) according to the manufacturer’s instructions. Following a clarification by centrifugation at 3000 rpm for 10 minutes, nucleic acids were extracted using the MagMAX Pathogen RNA/DNA Kit (Ambion) according to the manufacturer’s instructions.

cDNA synthesis was then performed using SuperScript™ IV First-Strand Synthesis System (SSIV) (ThermoFisher) according to the manufacturer’s recommendation. A total of 11 uL of extracted total nucleic acid was mixed with dNTPS (10 mM) and a tagged random nonamer primer (40 uM) (GTT TCC CAG TCA CGA TAN NNN NNN NN). Samples were incubated at 65 °C for 5 minutes, and then placed on ice for 1 minute. A reagent mixture of 5x SSIV Buffer, Ribonuclease Inhibitor (40 U/μL), DTT (100 mM) and SuperScript™ IV Reverse Transcriptase was then added. The samples were incubated for 10 minutes at 23 °C, 10 minutes at 50 °C and 10 minutes at 80 °C.

Second strand synthesis was performed using Sequenase Version 2.0 DNA Polymerase (ThermoFisher) according to the manufacturer’s recommendation. The first strand synthesis product was incubated with 10 uL of Sequenase Version 2.0 DNA Polymerase diluted in 5x reaction buffer and nuclease free water. Samples were then heated to 37 °C over five minutes and incubated at 37 °C for 12 minutes, followed by 2 minutes at 95 °C. Samples were then cooled to 10 °C and 1.2 uL of Sequenase DNA polymerase in dilution buffer was added. Samples were again ramped to 37 °C over five minutes and incubated at 37 °C for 12 minutes, followed by 8 minutes at 95 °C. A total of 6 uL of the second strand synthesis product was then used as template for amplification. AccuPrime™ *Taq* DNA Polymerase (Thermofisher) was mixed with 10X AccuPrime™ PCR Buffer I, nuclease free water and a primer for the nonomer’s tag (100 uM). 30 cycles of PCR were then performed with the following parameters: 30 seconds at 94 °C, 30 seconds at 40 °C, 30 seconds at 50 °C and 1 minute at 72 °C. cDNA/DNA mixtures were then cleaned with Genomic DNA Clean & Concentrator columns (Zymo Research) and eluted in 20 mM Tris (ThermoFisher).

### Library preparation and sequencing

Sequence libraries were prepared with the KAPA HyperPlus library kit (Roche). Sequence library construction and capture were carried out according to Nimblegen’s SeqCap EZ HyperCap Workflow User’s Guide v1. Samples were pooled in equal amounts by weight prior to capture. Sequencing was performed on an Illumina Miseq instrument in the National Centre for Foreign Animal Disease biocontainment level 3 sequencing facility. A V2 flow cell was used with a 500 cycle reagent cartridge (Illumina).

### 5′ Race and Sanger sequencing

5′ RACE was used to obtain the missing leader sequence (52 bp). The SMARTer 5′ RACE and 3′ RACE kit (Takarabio) was used according to the kit instructions. The gene specific primer used for 5′ RACE was TCAGCTACAGTAGAGGGAGATGTCATAGGTGC. For Sanger sequencing, amplicons was performed using KAPA HiFi HotStart ReadyMixPCR Kit (KAPABiosystems). The primers CTAAAGAGAAGGTGGACACTGGT and CTAAGAATGCGAACTTCACAGAGC were used to amplify the gene 4b homologue region. The primers GTTGTTGTGTTACAAGGCAAGGG and GGATTATGATCAAACCATGAACCTGG were used to amplify the NSP 10/12 region. Cycling conditions used to generate amplicon for Sanger sequencing were: 1 cycle: 95 °C for 3 minutes, 40 cycles: 98 °C for 20 seconds, 65 °C for 15 seconds, 72 °C for 2.5 minutes, and 1 cycle: 72 °C for 3 minutes. Amplicons were cleaned using AMPure XP beads (Beckman Coulter) according to the manufacturer’s directions. Sanger sequencing was performed on the ABI Genetic Analyzer 3130XL platform using the BigDye Terminator v3.1 Cycle Sequencing Kit (Applied Biosystems) according to the user manual.

### Bioinformatics

Read quality was assessed using FastQC and trimmed using Trimmamatic^27^ (Version 0.36). Host reads were then filtered with RAMBO- K, using the only complete genome of a goose species (*Anser cygnoides*) currently available and DCoV^28^. The near complete genome sequence of CGCoV was assembled from NGS derived sequences from a cloacal swab of one Canada goose using SPAdes^29^. Sanger reads were aligned to the draft genome in Geneious^TM^ (Biomatters, v 9.1.8). Annotations were performed using Geneious and protein domains were identified using PFAM^30^. The Canada goose coronavirus genome is available under accession number MK359255 on NCBI.
